# The Impact of RIPK1 Kinase Inhibition on Atherogenesis: A Genetic and a Pharmacological Approach

**DOI:** 10.3390/biomedicines10051016

**Published:** 2022-04-28

**Authors:** Pauline Puylaert, Isabelle Coornaert, Cédric H. G. Neutel, Yves Dondelinger, Tom Delanghe, Mathieu J. M. Bertrand, Pieter-Jan Guns, Guido R. Y. De Meyer, Wim Martinet

**Affiliations:** 1 Laboratory of Physiopharmacology, Faculty of Pharmaceutical, Biomedical and Veterinary Sciences, University of Antwerp, Universiteitsplein 1, 2610 Antwerp, Belgium; pauline.puylaert@uantwerpen.be (P.P.); isabelle.coornaert@uantwerpen.be (I.C.); guido.demeyer@uantwerpen.be (G.R.Y.D.M.); 2 Laboratory of Physiopharmacology, Faculty of Medicine and Health Sciences, University of Antwerp, Universiteitsplein 1, 2610 Antwerp, Belgium; cedric.neutel@uantwerpen.be (C.H.G.N.); pieter-jan.guns@uantwerpen.be (P.-J.G.); 3 VIB Center for Inflammation Research, 9052 Ghent, Belgium; yves.dondelinger@irc.vib-ugent.be (Y.D.); tom.delanghe@irc.vib-ugent.be (T.D.); mathieu.bertrand@irc.vib-ugent.be (M.J.M.B.); 4 Department of Biomedical Molecular Biology, Ghent University, 9052 Ghent, Belgium

**Keywords:** RIPK1, atherosclerosis, necroptosis

## Abstract

RIPK1 (receptor-interacting serine/threonine-protein kinase 1) enzymatic activity drives both apoptosis and necroptosis, a regulated form of necrosis. Because necroptosis is involved in necrotic core development in atherosclerotic plaques, we investigated the effects of a RIPK1^S25D/S25D^ mutation, which prevents activation of RIPK1 kinase, on atherogenesis in ApoE^−/−^ mice. After 16 weeks of western-type diet (WD), atherosclerotic plaques from ApoE^−/−^ RIPK1^S25D/S25D^ mice were significantly larger compared to ApoE^−/−^ RIPK1^+/+^ mice (167 ± 34 vs. 78 ± 18 × 10^3^ µm^2^, *p* = 0.01). Cell numbers (350 ± 34 vs. 154 ± 33 nuclei) and deposition of glycosaminoglycans (Alcian blue: 31 ± 6 vs. 14 ± 4%, *p* = 0.023) were increased in plaques from ApoE^−/−^ RIPK1^S25D/S25D^ mice while macrophage content (Mac3: 2.3 ± 0.4 vs. 9.8 ± 2.4%, *p* = 0.012) was decreased. Plaque apoptosis was not different between both groups. In contrast, pharmacological inhibition of RIPK1 kinase with GSK’547 (10 mg/kg BW/day) in ApoE^−/−^ Fbn1^C1039G+/−^ mice, a model of advanced atherosclerosis, did not alter plaque size after 20 weeks WD, but induced apoptosis (TUNEL: 136 ± 20 vs. 62 ± 9 cells/mm^2^, *p* = 0.004). In conclusion, inhibition of RIPK1 kinase activity accelerated plaque progression in ApoE^−/−^ RIPK1^S25D/S25D^ mice and induced apoptosis in GSK’547-treated ApoE^−/−^ Fbn1^C1039G+/−^ mice. Thus, without directly comparing the genetic and pharmacological studies, it can be concluded that targeting RIPK1 kinase activity does not limit atherogenesis.

## 1. Introduction

Rupture of vulnerable atherosclerotic plaques leads to acute clinical complications such as myocardial infarction or stroke and accounts for about 85% of cardiovascular deaths [1]. One of the main characteristics of a vulnerable plaque is the presence of a large necrotic core. In almost 90% of ruptured human plaques, the necrotic core comprises >10% of the plaque area while almost 65% of ruptured plaques contain a necrotic core that occupies >25% of the plaque [2]. Recent studies have demonstrated that regulated necrosis, in particular necroptosis, plays a significant role in the formation of a necrotic core during atherosclerotic plaque development [3,4,5,6,7,8,9].

Necroptosis, in contrast to apoptosis, leads to a pro-inflammatory state and can be induced by various stimuli, including oxidized LDL and TNFα [4,10]. Upon TNFα stimulation, the fate of the cell towards survival, apoptotic, or necroptotic pathways is dependent on the ubiquitination and phosphorylation profile of receptor-interacting serine/threonine protein kinase 1 (RIPK1) [11]. When inactive, RIPK1 serves as a scaffold for pro-survival pathways, while in an active state it can act as a kinase in RIPK1-dependent apoptosis or, if proapoptotic caspase 8 is inhibited, in necroptosis [12,13,14,15,16,17]. The latter is characterized by phosphorylation events of RIPK1 and RIPK3, which ultimately lead to the recruitment and phosphorylation of mixed lineage kinase domain-like pseudokinase (MLKL) [18,19,20,21]. Activated MLKL forms homo-oligomers, which migrate towards the plasma membrane to promote membrane rupture and cell lysis [22,23].

A recent study showed that the administration of anti-sense oligonucleotides (ASOs) directed against MLKL reduces plaque necrosis in ApoE^−/−^ mice by ≈ 50% [5]. However, contradictory results have been reported after the deletion of the key necroptosis executioner RIPK3 [3,9,24]. According to Lin et al. [3], gene deletion of RIPK3 in LDLr^−/−^ mice results in a reduced lesion size and smaller necrotic cores in advanced plaques. More recently, another study reported that loss of RIPK3 in smooth muscle cells (or globally) does not affect lesion size and necrotic core, whereas RIPK3 deletion in macrophages or endothelial cells exacerbates atherosclerosis [24]. Interestingly, high RIPK1 expression levels are reported in human atherosclerotic lesions [8,25]. Both silencing of RIPK1 with ASOs and myeloid-specific RIPK1 gene deletion in ApoE^−/−^ mice slow down plaque progression [4,8]. However, the latter approach also results in impaired NF-κB signaling and a switch to RIPK1-independent cell death, eventually leading to a similar lesion size and necrotic core in more advanced lesions as compared to control mice [7]. These findings demonstrate the complex involvement of RIPK1, either as a scaffold in pro-survival pathways such as NF-κB signaling or as an active kinase in RIPK1-dependent necrotic cell death, making it difficult to draw conclusions about the precise role of RIPK1 in plaque development.

To better understand the role of RIPK1 kinase activity in advanced atherosclerosis, we crossbred ApoE^−/−^ mice with RIPK1^S25D/S25D^ mice carrying a RIPK1 point mutation that converts serine 25 into aspartic acid (S25D). Serine 25, when phosphorylated, serves as a brake preventing engagement of RIPK1 in cell death pathways. Aspartic acid mimics phosphorylated serine 25, resulting in inhibition of RIPK1-dependent cell death without affecting its pro-survival scaffolding function [13]. Apart from the phospho-mimetic S25D mutation, the kinase activity of RIPK1 can be inhibited using pharmacological inhibitors. Nec-1 is a well-known RIPK1 kinase inhibitor but displays off-target effects and poor metabolic stability. Nec-1s is a stable Nec-1 analog that reduces the lesion area and necrotic core in ApoE^−/−^ mice [4]; however, its in vivo selectivity and potency remain poorly studied. Novel RIPK1 kinase inhibitors have recently been developed with increased potency and improved pharmacokinetic profiles as compared to Nec-1s [25,26]. For example, GSK’547 potently binds to RIPK1 with exquisite kinase specificity (complete selectivity against >450 off-target kinases) and displays a high oral bioavailability and >90% RIPK1 inhibition for sustained periods in mice [26]. By administering GSK’547, we aimed to evaluate the drugability of RIPK1 kinase in ApoE^−/−^ Fbn1^C1039G+/−^ mice, which is a unique model of advanced human-like atherosclerosis [27,28].

## 2. Materials and Methods

### 2.1. Mice

Standard ApoE^−/−^ mice (Jackson Laboratory, Bar Harbor, ME, USA, 002052) were crossbred with C57BL/6 mice containing a phospho-mimetic S25D mutation in the RIPK1 coding sequence that strongly suppresses RIPK1 kinase activity [13]. The resulting ApoE^−/−^ RIPK1^S25D/S25D^ mice and ApoE^−/−^ RIPK1^+/+^ controls (all female, 6–8 weeks old) were fed a western-type diet (WD; C1000 supplemented with 20% milkfat and 0.15% cholesterol, Altromin, Lage, Germany) for 16 weeks to induce plaque formation. Only female mice were used because plaque formation is more severe in females as compared to males in mouse models of atherosclerosis after 16 weeks WD [29,30]. Previous experiments in our research group confirmed that established plaques are present at this timepoint and not much is gained, regarding plaque phenotype, by feeding WD for longer periods in ApoE^−/−^ mice. The animals were housed in a temperature-controlled room with a 12 h light/dark cycle and had free access to water and food. After 16 weeks WD, an overdose of sodium pentobarbital (250 mg/kg, i.p.) was administered and blood samples were collected via the retro-orbital plexus. Plasma levels of total cholesterol were measured using a commercially available kit (Randox Laboratories, Crumlin, UK). Non-responding mice to WD (plasma cholesterol <400 mg/dL) were excluded.

In another series of experiments, female ApoE^−/−^ mice with a heterozygous mutation (C1039G+/−) in the fibrillin 1 (Fbn1) gene were fed either WD or WD supplemented with RIPK1 inhibitor GSK’547 (10 mg/kg BW/day) starting at the age of 6–8 weeks and sacrificed after 20 weeks WD. They were fed WD for 20 weeks because it was previously reported that this induces an advanced plaque phenotype in ApoE^−/−^ Fbn1^C1039G+/−^ mice [27,28]. The daily dosage of GSK’547 was based on previous food-based GSK’547 studies in mice [26,31]. For diets containing 10 mg/kg BW/day GSK’547, the plasma concentration of the drug varies between 10 ng/mL (at the trough of the eating cycle) and 100 ng/mL (at peak of the eating cycle), which provides 75% to 97% inhibition in vivo, respectively. For WD supplemented with 10 mg/kg BW/day GSK’547, plasma levels of over 100 ng/mL and 150 ng/mL were reported after 2 and 4 weeks, respectively, which is well above the IC50 of 13 ng/mL (32 nM) [31]. Female ApoE^−/−^ Fbn1^C1039G+/−^ mice are essential because, similar to humans, the Fbn1 mutation frequently causes aortic dissection (and mortality) in male ApoE^−/−^ Fbn1^C1039G+/−^ mice and not in female ApoE^−/−^ Fbn1^C1039G+/−^ mice [28].

Blood leukocyte subsets were analyzed by flow cytometry as previously described [32]. All experiments were ethically reviewed by the Ethical Committee of the University of Antwerp (Code 2018-01) and carried out in accordance with European Directive 2010/63/EEC.

### 2.2. Echocardiography

Echocardiograms were performed on anesthetized ApoE^−/−^ Fbn1^C1039G+/−^ mice (isoflurane, 4% for induction and 2% for maintenance) at the start (0 weeks WD) and at the end of the GSK’547 study (20 weeks WD) using a VEVO2100 (VisualSonics, Amsterdam, The Netherlands), equipped with a 25 MHz transducer. Body temperature was maintained at 36–38 °C and heart rate at 500 ± 50 beats/min. To study arterial stiffness, pulse wave velocity (PWV) was determined in the abdominal aorta using a 24 MHz transducer, as previously described [33]. Briefly, the aortic diameter (D) was measured on 700 frames-per-second B-mode images of the abdominal aorta in EKV mode. Subsequently, the aortic flow velocity (V) was determined by pulse wave Doppler tracing. PWV was then calculated via the ln(D)-V loop method using MathLab v2014 software (MathWorks, Natick, MA, USA).

### 2.3. Histological Analyses

The proximal ascending aorta and brachiocephalic artery were fixed in 4% formaldehyde (pH 7.4) for 24 h, dehydrated overnight in 60% isopropanol, and subsequently embedded in paraffin. The proximal ascending aorta was marked on the distal arch end and the brachiocephalic artery on the distal carotid end to ensure that they were always cut on the proximal side. Serial cross-sections (5 µm) of the proximal parts of the proximal ascending aorta and the brachiocephalic artery were prepared at random for histological analyses. Although plaque formation was also observed in other vascular beds (e.g., aorta arch, carotids, and coronary arteries), we focused on the proximal ascending aorta and brachiocephalic artery because here plaque formation was observed in >90% of the mice, in both ApoE^−/−^ and ApoE^−/−^ Fbn1^C1039G+/−^ mice. Moreover, plaques in the brachiocephalic artery enter an advanced, human-like stage more rapidly [34,35]. Atherosclerotic plaque size, necrotic core area (defined as acellular areas with a threshold of 3000 µm^2^), internal elastic lamina (IEL) area, thickness of the tunica media, and degree of stenosis were analyzed on hematoxylin/eosin (H&E)-stained sections. The IEL was manually delineated, the IEL perimeter was measured, and subsequently, the IEL area was calculated using formulas for circle perimeter (2 *pi *r) and area (pi *r^2^). The external elastic lamina (EEL) was manually delineated, the EEL perimeter was measured, and subsequently, the media thickness was calculated by subtracting the IEL radius from the EEL radius. Collagen and glycosaminoglycan content was determined on Sirius red and Alcian blue-stained sections, respectively. Apoptosis was analyzed using the ApopTag Plus Peroxidase In Situ Apoptosis Kit (Millipore, Burlington, VT, USA, S7101). For immunohistochemistry, the following antibodies were used: anti-Mac3 (BD Pharmingen, San Diego, CA, USA, 550292), anti-α-smooth muscle actin (α-SMA, Sigma-Aldrich, St. Louis, MO, USA, A2547), and anti-cleaved caspase 3 (Cell Signaling, Danvers, TX, USA, 9661). Images were acquired with an Olympus BX43 microscope, which was calibrated for each magnification. Plaque size was measured based on pixels per µm, which was determined during the calibration of the microscope. Per mouse, one section was analyzed. Plaques and tunica media of the vessel wall were manually delineated in ImageJ software (National Institutes of Health, Bethesda, MD, USA) to establish the region of interest (ROI). Further analyses within the ROIs were performed using color thresholding or manual counting (apoptotic cells).

### 2.4. Cell Culture

Bone marrow-derived macrophages (BMDMs) were harvested by flushing bone marrow of the femur with a 25 Gauge needle and heparinized (10 IU/mL) RPMI 1640 medium (Gibco Life Technology, Merelbeke, Belgium). After washing and filtration, cells were cultured in RPMI 1640 medium supplemented with Glutamax (Gibco Life Technology) and 15% L929 cell-conditioned medium (LCCM) containing monocyte colony-stimulating factor (M-CSF) for 7 days in 95% air/5% CO_2_ until 80–90% confluency was reached. To induce necroptosis, BMDMs were treated with 50 ng/mL lipopolysaccharide (LPS, Sigma-Aldrich) and 20 µM zVAD-fmk (Enzo Life Sciences, Brussels, Belgium, ALX-260-020) for 18 h. Necrosis was evaluated by labeling cells with 1 µg/mL propidium iodide (PI, Molecular Probes, Eugene, OR, USA) and 10 µg/mL Hoechst (Life Technologies, Carlsbad, CA, USA), followed by visualization of PI/Hoechst-labeled cells using a Celena S digital imaging system (Logos Biosystems, Dongan-gu, Anyang-si, Korea).

### 2.5. Western Blotting

Tissues were homogenized in RIPA buffer containing protease and phosphatase inhibitors. Protein concentrations were determined using the BCA method. Samples were then 1:1 diluted in Laemmli sample buffer (Bio-Rad, Hercules, CA, USA) containing 5% β-mercaptoethanol (Sigma-Aldrich) and heat-denatured for 5 min at 100 °C. Samples were loaded on Bolt 4–12% Bis-Tris gels (Invitrogen, Waltham, MA, USA) and after electrophoresis transferred to Immobilon-FL PVDF membranes (Millipore) according to standard procedures. Subsequently, membranes were blocked for one hour in Odyssey Li-COR blocking buffer. After blocking, membranes were probed with primary antibodies diluted in Odyssey Li-COR blocking buffer followed by 1 h incubation with IRDye-labeled secondary antibodies at room temperature. Membranes were visualized with an Odyssey SA infrared imaging system (Li-COR Biosciences, Lincoln, NE, USA).

The following primary antibodies were used: mouse anti-β-actin (Abcam, Cambridge, UK, ab8226), mouse anti-RIPK1 (BD Transduction Laboratories, Franklin Lakes, NJ, USA, 610459), rabbit anti-phospho-RIPK1 (Cell Signaling, 31122), rabbit anti-RIPK3 (Abcam, ab62344), rabbit anti-phospho-RIPK3 (Abcam, ab195117), rabbit anti-MLKL (Abcam, ab194699), rabbit anti-phospho-MLKL (Abcam, ab196436), rabbit anti-IκBα (Cell Signaling, 9242), rabbit anti-phospho-IκBα (Cell Signaling, 2859), rabbit anti-NF-κB (Cell Signaling, 4764), rabbit anti-phospho-NF-κB (Cell Signaling, 3031), rabbit anti-p38 (Cell Signaling, 9212), and rabbit anti-phospho-p38 (Cell Signaling, 9211). IRDye-labeled secondary antibodies (IgG926-32211 (goat anti-rabbit); IgG926-68070 (goat anti-mouse)) were purchased from Li-COR Biosciences.

### 2.6. Statistical Analyses

Statistical analyses were performed using GraphPad Prism 9 and SPSS software (version 27, SPSS Inc., Chicago, IL, USA). All data were expressed as the mean ± SEM, dots represent *n* samples from independent experiments or individual mice. Statistical tests are specified in the text and figure legends. Differences were considered significant when *p* < 0.05.

## 3. Results

### 3.1. RIPK1^S25D/S25D^ BMDMs Are Protected against Necroptosis

**Figure 1:**
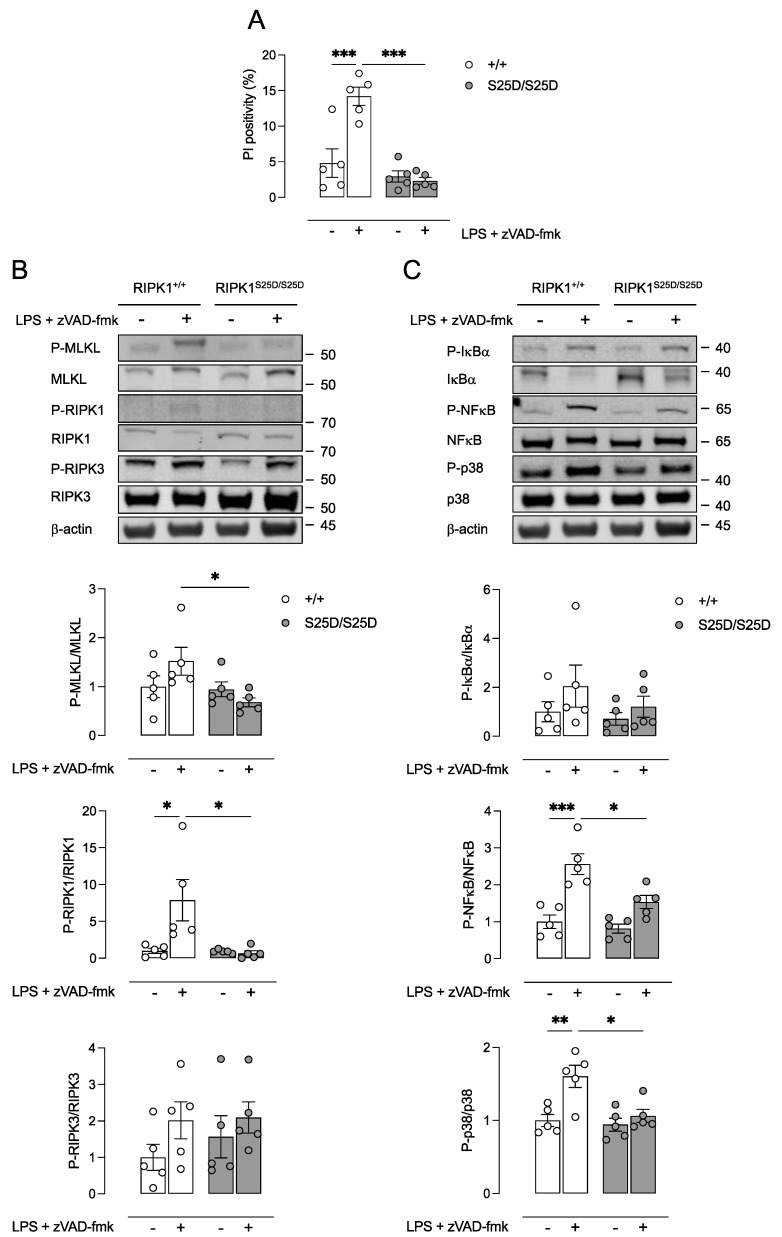
RIPK1^S25D/S25D^ macrophages are protected against LPS/zVAD-fmk-induced necroptosis. Bone marrow-derived macrophages (BMDMs) were isolated from RIPK1^+/+^ and RIPK1^S25D/S25D^ mice and treated with 50 ng/mL LPS and 20 µM zVAD-fmk for 18 h. (**A**) Cell death was measured using propidium iodide (PI) labeling. (**B**,**C**) Western blot analysis on cell lysates of necroptosis proteins (RIPK1, RIPK3, MLKL) as well as proteins associated with the scaffolding function of RIPK1 regulating inflammatory signaling (NF-κB, IκBα, p38). * *p* < 0.05, ** *p* < 0.01, *** *p* < 0.001 (two-way ANOVA followed by Tukey’s post hoc test, *n* = 5 independent experiments).

We previously reported that RIPK1 is mainly expressed in macrophage-rich areas of atherosclerotic plaques [7]. To validate the inhibition of RIPK1 kinase-dependent necroptosis in macrophages, BMDMs were isolated from RIPK1^+/+^ and RIPK1^S25D/S25D^ mice. Next, isolated cells were treated with LPS in combination with the pan-caspase inhibitor zVAD-fmk to induce necroptosis. Significant induction of cell death was only observed in RIPK1^+/+^ BMDMs while RIPK1^S25D/S25D^ BMDMs were protected against LPS/zVAD-fmk treatment (Figure 1A). Moreover, expression of phosphorylated MLKL (P-MLKL) and phosphorylated RIPK1 (P-RIPK1) were significantly increased in RIPK1^+/+^ BMDMs after LPS/zVAD-fmk treatment, though unchanged in RIPK1^S25D/S25D^ BMDMs (Figure 1B). These results indicate that necroptosis was efficiently blocked in vitro in BMDMs carrying the S25D mutation as reported previously [13]. Importantly, P-RIPK1 could be detected in LPS/zVAD-fmk-treated RIPK1^+/+^ BMDMs but showed very weak bands in untreated RIPK1^+/+^ BMDMs and in RIPK1^S25D/S25D^ BMDMs. Therefore, the calculated P-RIPK1/RIPK1 ratios should be interpreted cautiously. For RIPK3 no significant changes in the expression level nor the phosphorylation were observed in RIPK1^S25D/S25D^ BMDMs (Figure 1B). Interestingly, the expression of the NF-κB inhibitor IκBα, but not phosphorylated IκBα, was increased in RIPK1^S25D/S25D^ BMDMs, and consequently, a decreased expression of phosphorylated NF-κB was observed, as compared to RIPK1^+/+^ BMDMs (Figure 1C). In addition, the phosphorylation of p38 was decreased in RIPK1^S25D/S25D^ BMDMs as compared to RIPK1^+/+^ BMDMs (Figure 1C).

### 3.2. ApoE^−/−^ RIPK1^S25D/S25D^ Mice Develop Larger Plaques with Increased Deposition of Extracellular Matrix Components

**Figure 2:**
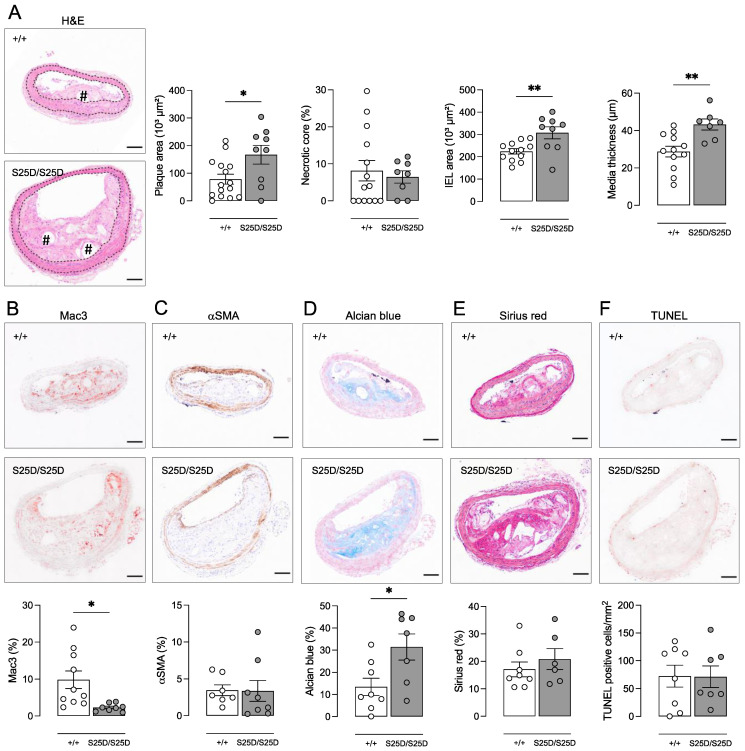
ApoE^−/−^ RIPK1^S25D/S25D^ mice show positive vascular remodeling and larger plaques with increased deposition of extracellular matrix components. ApoE^−/−^ RIPK1^+/+^ and ApoE^−/−^ RIPK1^S25D/S25D^ mice were fed a WD for 16 weeks. Sections of the brachiocephalic artery were stained with (**A**) hematoxylin/eosin to quantify plaque size, necrotic core (# hash signs) and vessel properties (dotted lines delineate the media), (**B**) anti-Mac3 to determine macrophage content, (**C**) anti-α-smooth muscle actin (αSMA) to determine vascular smooth muscle cell content, (**D**) Alcian blue to quantify glycosaminoglycans, (**E**) Sirius red to quantify total collagen and (**F**) TUNEL to count apoptotic cells. * *p* < 0.05, ** *p* < 0.01 (independent samples *t*-test, *n* = 7–15 mice per group). Scale bar = 100 µm. Representative images are shown.

To evaluate the effect of RIPK1 kinase inhibition on atherosclerotic plaque development, ApoE^−/−^ RIPK1^+/+^ and ApoE^−/−^ RIPK1^S25D/S25D^ mice were fed a western-type diet (WD) for 16 weeks. Body weight (24.6 ± 0.9 vs. 25.0 ± 0.8 g; independent samples *t*-test, *p* > 0.05), plasma cholesterol (609 ± 23 vs. 598 ± 24.9 mg/dL; independent samples *t*-test, *p* > 0.05) and circulating leukocyte subsets (Supplementary Figure S1) were not different between ApoE^−/−^ RIPK1^+/+^ and ApoE^−/−^ RIPK1^S25D/S25D^ mice after 16 weeks WD. ApoE^−/−^ RIPK1^S25D/S25D^ mice developed significantly larger plaques in the brachiocephalic artery (167 ± 34 vs. 78 ± 18 × 10^3^ µm^2^; independent samples *t*-test, *p* = 0.010), albeit without an increase in the relative necrotic core area (Figure 2A). An increase in the number of nuclei was observed in plaques of ApoE^−/−^ RIPK1^S25D/S25D^ mice (350 ± 34 vs. 154 ± 33 nuclei; Mann–Whitney test *p* = 0.002; data not shown), while the macrophage content was significantly decreased (2.3 ± 0.4 vs. 9.8 ± 2.4% Mac3; independent samples *t*-test, *p* = 0.012) as compared to ApoE^−/−^ RIPK1^+/+^ controls (Figure 2B). The relative expression of α-smooth muscle actin, used as a vascular smooth muscle cell marker, was not different between both groups (Figure 2C). However, the production of extracellular matrix was increased in plaques of ApoE^−/−^ RIPK1^S25D/S25D^ mice as glycosaminoglycans were almost tripled (31.4 ± 5.9 vs. 13.5 ± 3.9% Alcian blue; independent samples *t*-test, *p* = 0.023) in plaques of ApoE^−/−^ RIPK1^S25D/S25D^ mice versus ApoE^−/−^ RIPK1^+/+^ controls (Figure 2D). The area occupied by collagen increased with increased plaque size in ApoE^−/−^ RIPK1^S25D/S25D^ mice (37.7 ± 5.4 vs. 13.5 ± 3.3 × 10^3^ µm^2^; independent samples *t*-test, *p* = 0.001). However, this increase was not present when calculating total collagen content relative to plaque size (Figure 2E).

Because necrotic core formation was not altered in plaques of ApoE^−/−^ RIPK1^S25D/S25D^, the expression of apoptosis and necroptosis markers was evaluated in plaques of ApoE^−/−^ RIPK1^S25D/S25D^ and ApoE^−/−^ RIPK1^+/+^ mice. Both immunohistochemical analysis of cleaved caspase 3 and TUNEL labeling did not show any differences in the frequency of apoptosis between both groups (Figure 2F). Western blot analyses of P-MLKL and RIPK1 did not reveal differences either (data not shown).

As plaques were significantly larger in ApoE^−/−^ RIPK1^S25D/S25D^ mice, while stenosis was (borderline) not significantly different (48.6 ± 8.7 vs. 26.3 ± 7.0% stenosis; independent samples *t*-test, *p* = 0.055), the vessel diameter and wall of the brachiocephalic artery were also evaluated. Interestingly, the internal elastic lamina (IEL) area was significantly increased in ApoE^−/−^ RIPK1^S25D/S25D^ mice as compared to ApoE^−/−^ RIPK1^+/+^ controls (Figure 2A), suggesting positive vascular remodeling. Moreover, the vessel media thickness was significantly elevated in ApoE^−/−^ RIPK1^S25D/S25D^ mice (Figure 2A) meaning that vessel remodeling is accompanied by hypertrophy. In line with these findings, the glycosaminoglycan content was significantly increased in the vessel media of ApoE^−/−^ RIPK1^S25D/S25D^ mice (4.3 ± 1.5 vs. 1.4 ± 0.4% Alcian blue; independent samples *t*-test, *p* = 0.04), similarly to what was observed in plaques, confirming extracellular matrix changes.

### 3.3. ApoE^−/−^ Fbn1^C1039G+/−^ Mice Can Be Used as a Tool to Study Necroptosis in Atherosclerosis

**Figure 3:**
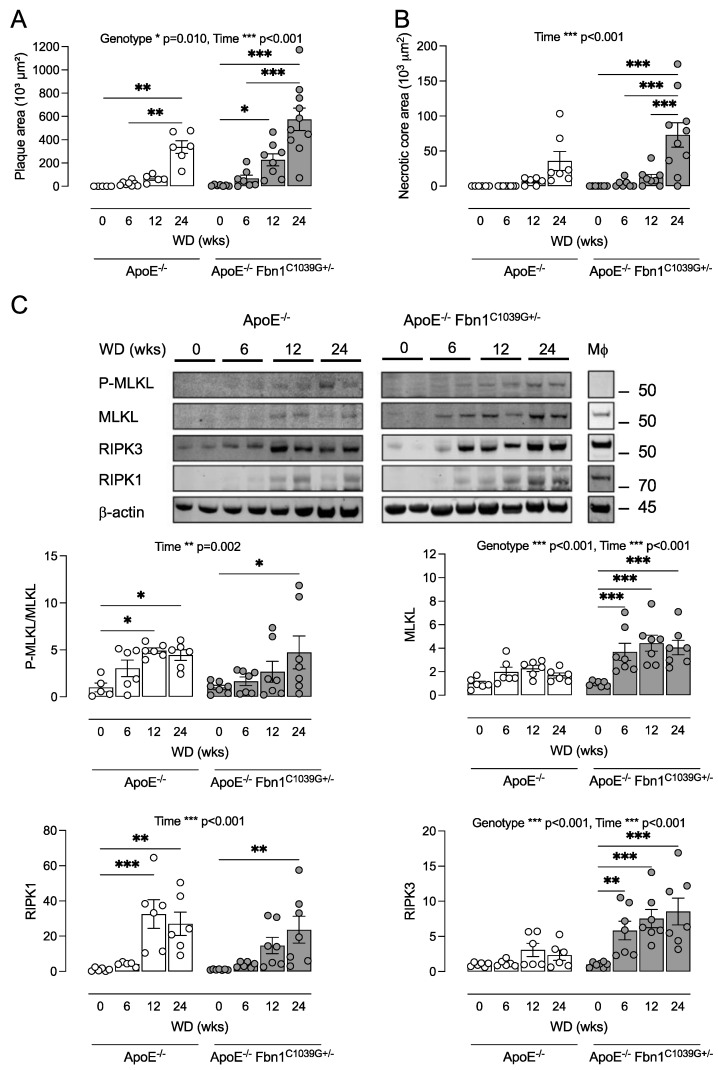
ApoE^−/−^ and ApoE^−/−^ Fbn1^C1039G+/−^ mice can be used as tools to study necroptosis in atherosclerosis. ApoE^−/−^ and ApoE^−/−^ Fbn1^C1039G+/−^ mice (genotype factor) were fed a western-type diet (WD) for 6, 12, and 24 weeks (time factor). Sections of the proximal ascending aorta were stained with hematoxylin/eosin to measure (**A**) plaque size and (**B**) necrotic core area. (**C**) Expression of necroptosis proteins (MLKL, RIPK1, RIPK3) was analyzed via Western blotting in plaque lysates of the aortic arch as well as in bone marrow-derived macrophages (Mφ) of wild-type mice. * *p* < 0.05, ** *p* < 0.01, *** *p* < 0.001 vs. 0 weeks WD (two-way ANOVA followed by Dunnett’s post hoc test between timepoints per genotype, *n* = 6–10 mice per group).

The ApoE^−/−^ Fbn1^C1039G+/−^ mouse is a recently developed model with a heterozygous mutation (C1039G+/−) in the fibrillin 1 gene (Fbn1), which results in fragmentation of the elastin fibers in the media of the vessel wall and increased arterial stiffness. Moreover, ApoE^−/−^ Fbn1^C1039G+/−^ mice develop advanced atherosclerotic lesions with human-like features such as large necrotic cores, intraplaque (IP) neovessels, IP hemorrhages, IP inflammation, and spontaneous plaque rupture leading to myocardial infarction and stroke [27,28]. Because necroptosis in experimental atherosclerosis has so far only been studied in classical mouse models such as the ApoE^−/−^ or LDLr^−/−^ mouse [3,4,5,7,8,9,24], we analyzed the expression of necroptosis proteins in plaques of ApoE^−/−^ and ApoE^−/−^ Fbn1^C1039G+/−^ mice. To this end, both ApoE^−/−^ and ApoE^−/−^ Fbn1^C1039G+/−^ mice were fed a WD for 6–24 weeks. Compared to ApoE^−/−^ mice, ApoE Fbn1^C1039G+/−^ mice developed 50% larger plaques with a significantly larger necrotic core after 24 weeks WD (two-way ANOVA followed by Sidak’s post hoc test between genotypes per timepoint, *p* = 0.03) as compared to ApoE^−/−^ mice (Figure 3A,B). Necrotic core formation in ApoE^−/−^ Fbn1^C1039G+/−^ mice started after 6 weeks WD, whereas in ApoE^−/−^ mice the necrotic core was only detectable after 12 weeks WD (Figure 3B). At baseline (0 weeks WD), very low levels of RIPK1, RIPK3, and MLKL were observed in the atherosclerosis-prone aortic arch of both ApoE^−/−^ and ApoE^−/−^ Fbn1^C1039G+/−^ mice but the expression levels increased during plaque progression (Figure 3C). In plaques of ApoE^−/−^ Fbn1^C1039G+/−^ mice the expression of RIPK3 and MLKL started to increase significantly after 6 weeks WD, in contrast to plaques of ApoE^−/−^ mice where no significant increases were observed compared to 0 weeks WD. Accordingly, plaques of ApoE^−/−^ Fbn1^C1039G+/−^ mice expressed significantly higher levels of MLKL and RIPK3 as compared to ApoE^−/−^ mice. The expression of RIPK1 was significantly increased after 24 weeks WD in plaques of ApoE^−/−^ Fbn1^C1039G+/−^ mice and after 12 weeks WD in plaques of ApoE^−/−^ mice. No significant difference in RIPK1 expression was observed between the two genotypes. Because macrophages express RIPK1, RIPK3, and MLKL, the elevated expression levels of these markers may be caused by increased macrophage infiltration during plaque formation. However, a significant increase in the P-MLKL/MLKL ratio was also observed in plaques of both ApoE^−/−^ and ApoE^−/−^ Fbn1^C1039G+/−^ mice (Figure 3C), demonstrating that MLKL is actively phosphorylated in the plaques and suggestive of necroptosis initiation. Despite significantly higher levels of MLKL in ApoE^−/−^ Fbn1^C1039G+/−^ mice as compared to ApoE^−/−^ mice, no significant difference in the P-MLKL/MLKL ratio was observed.

### 3.4. Pharmacological Inhibition of RIPK1 with GSK’547 Does Not Alter Plaque Size and Composition in ApoE^−/−^ Fbn1^C1039G+/−^ Mice

**Figure 4:**
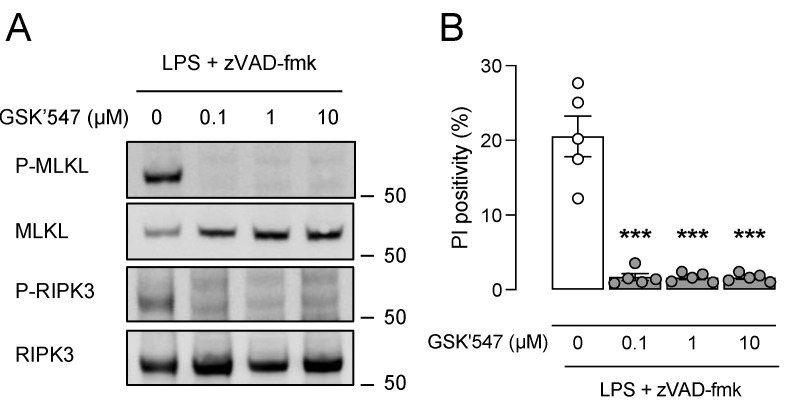
GSK’547 protects macrophages from LPS/zVAD-fmk-induced necroptosis. Bone marrow-derived macrophages (BMDMs) were isolated from wild-type mice and treated with 50 ng/mL LPS and 20 µM zVAD-fmk in the presence or absence of GSK’547 for 18 h. (**A**) Western blot analysis of necroptosis proteins (MLKL, RIPK3) on cell lysates. (**B**) Cell death was measured using propidium iodide (PI) labeling. *** *p* < 0.001 vs. 0 µM GSK’547 (one-way ANOVA followed by Dunnett’s post hoc test, *n* = 5 independent experiments).

**Figure 5:**
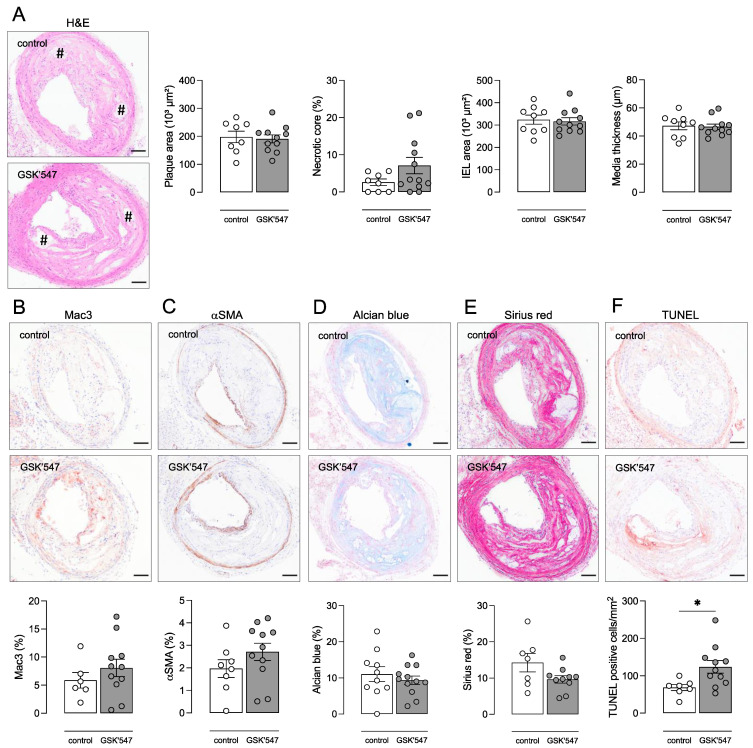
GSK’547 does not alter plaque size and composition in ApoE^−/−^ Fbn1^C1039G+/−^ mice but increases apoptosis. ApoE^−/−^ Fbn1^C1039G+/−^ mice were fed a WD supplemented with GSK’547 (10 mg/kg BW/day) for 20 weeks. Sections of the brachiocephalic artery were stained with (**A**) hematoxylin/eosin to measure plaque size, necrotic core (# hash signs), and vessel properties, (**B**) anti-Mac3 to determine macrophage content, (**C**) anti-α-smooth muscle actin (αSMA) to determine vascular smooth muscle cell content, (**D**) Alcian blue to quantify glycosaminoglycans, (**E**) Sirius red to quantify total collagen, and (**F**) TUNEL to count apoptotic cells. * *p* < 0.05 (independent samples *t*-test, *n* = 8–12 mice per group). Scale bar = 100 µm. Representative images are shown.

The ApoE^−/−^ Fbn1^C1039G+/−^ mouse model was applied to examine the effects of pharmacological RIPK1 kinase inhibition on advanced atherosclerotic plaques. In vitro experiments showed that the RIPK1 kinase inhibitor GSK’547 efficiently inhibited induction of cell death and phosphorylation of MLKL and RIPK3 in WT BMDMs after LPS/zVAD-fmk treatment, even at the lowest concentration tested (0.1 μM) (Figure 4). Therefore, ApoE^−/−^ Fbn1^C1039G+/−^ mice were fed a WD with or without GSK’547 (10 mg/kg BW/day) for 20 weeks to examine the effects of GSK’547 on the formation and composition of atherosclerotic plaques. Body weight (18.5 ± 0.5 vs. 19.7 ± 0.5 g; independent samples *t*-test, *p* = 0.052), plasma cholesterol (783 ± 71 vs. 799 ± 70 mg/dL; independent samples *t*-test, *p* > 0.05) and circulating leukocyte subsets (Supplementary Figure S2) were not different between GSK’547-treated mice and control mice. Since ApoE^−/−^ Fbn1^C1039G+/−^ mice are reported to develop increased arterial stiffness [27], the pulse wave velocity was analyzed using echocardiography after 0 and 20 weeks WD but showed no significant difference between GSK’547- and control-treated ApoE^−/−^ Fbn1^C1039G+/−^ mice (2.3 ± 0.4 vs. 2.6 ± 0.4 m/s at 0 weeks; 2.2 ± 0.2 vs. 2.2 ± 0.3 m/s after 20 weeks WD for controls and GSK’547-treated mice, respectively; two-way ANOVA followed by Bonferroni’s post hoc test, *p* > 0.05). Moreover, mortality was not different between GSK’547-treated mice (6 sudden deaths out of 22; 27%) and untreated controls (10 sudden deaths out of 20; 50%) after 16 weeks WD (log-rank test, *p* = 0.162). Along these lines, atherosclerotic plaque size was not significantly changed in the brachiocephalic artery of GSK’547-treated mice as compared to controls (191 ± 14 vs. 198 ± 20; independent samples *t*-test, *p* > 0.05), nor was the necrotic core area significantly different (Figure 5A). Immunohistochemical plaque analyses did not show any differences in macrophage (Figure 5B), vascular smooth muscle cell (Figure 5C), glycosaminoglycan (Figure 5D), and collagen (Figure 5E) content between both groups.

Interestingly, phosphorylation of MLKL was low in the aortic arch lysates of control mice and tended to increase in the GSK’547-treated group (3.6 ± 1.3 vs. 1.0 ± 0.2 P-MLKL/MLKL ratio (fold change), independent samples *t*-test *p* = 0.08), although not significantly. RIPK3 and p-RIPK3 levels were not different between the aortic arch lysates of GSK’547-treated mice and controls. However, levels of cleaved caspase 3 (0.49 ± 0.13 vs. 0.13 ± 0.04% cleaved caspase 3; independent samples *t*-test, *p* = 0.021; data not shown) as well as the number of TUNEL-positive cells per mm^2^ (124 ± 17 vs. 69 ± 8 cells/mm^2^; independent samples *t*-test, *p* = 0.023) significantly increased in plaques of GSK’547-treated mice as compared to controls (Figure 5F), suggesting the induction of apoptosis in plaques of GSK’547-treated mice.

## 4. Discussion

Despite recent findings showing that RIPK1 is a central driver of inflammation in atherosclerosis [4,7,8], the role of RIPK1 in the progression and destabilization of atherosclerotic plaques is not straightforward and is complicated by its dual nature, namely a scaffolding function (regulating pro-survival signaling and inflammatory gene expression) versus kinase activity (promoting cell death). In the present study, RIPK1^S25D/S25D^ mice containing an S > D mutation in the kinase domain of RIPK1 were crossbred with ApoE^−/−^ mice to investigate the role of RIPK1 kinase activity in experimental atherosclerosis. The S25 residue is phosphorylated by IKKs and the S > D mutation mimics this phosphorylation event. As a consequence, it strongly represses RIPK1 kinase activity both in vitro and in vivo [13]. We were able to confirm the inhibition of necroptosis in BMDMs isolated from RIPK1^S25D/S25D^ mice after treatment with LPS/zVAD-fmk. Interestingly, besides the inhibition of RIPK1 kinase activity, NF-κB and p38 activation were significantly decreased in RIPK1^S25D/S25D^ BMDMs as compared to BMDMs from RIPK1^+/+^ controls. This may be attributed to the fact that LPS, a TLR4 ligand, was used to induce necroptosis in vitro. After TLR4 binding, LPS induces Erk1/2 expression in a RIPK1 kinase-dependent but necroptosis-independent way. Erk1/2 in turn induces NF-κB- and cFos-dependent inflammatory gene expression [36]. It should be noted that induction of necroptosis in atherosclerotic plaques can be stimulated by other factors including atherogenic ligands such as oxLDL, through direct upregulation and activation of RIPK3 and MLKL, and by cytokine (TNFα) secretion associated with sterile inflammation [4].

Importantly, atherosclerosis was exacerbated in ApoE^−/−^ mice carrying the RIPK1^S25D/S25D^ mutation as compared to ApoE^−/−^ RIPK1^+/+^ controls. No differences in the necrotic core, cleavage of caspase 3, TUNEL, and necroptosis markers were observed, meaning that cell death was not significantly changed. This contrasts with two previous studies by Karunakaran et al. [4,8] in which ApoE^−/−^ mice were treated with RIPK1 kinase inhibitor Nec1s or antisense oligonucleotides (ASOs) against RIPK1. The authors reported that Nec1s reduced the plaque area in the aortic root and overall lesion burden in the aorta of ApoE^−/−^ mice [4]. Moreover, a significant decrease in the absolute P-MLKL-positive area was observed in aortic lesions of Nec-1s-treated mice, which could not be detected with immunohistochemistry in the present study. However, since Nec1s reduced the lesion area, it is unclear whether relative P-MLKL expression, and thus necroptosis, is truly inhibited in the plaques. Karunakaran et al. also reported that administration of RIPK1-specific ASOs resulted in a decreased, but not absent, expression of RIPK1 in ApoE^−/−^ mice [8]. In this way, a basal level of RIPK1 was maintained to preserve minimal pro-survival NF-κB signaling and to prevent spontaneous cell death. This is important because we previously reported that a complete RIPK1 knock-out in macrophages of ApoE^−/−^ mice results in impaired NF-κB signaling, increased apoptotic cell death, and plaque progression [7]. Interestingly, the authors reported that RIPK1-specific ASOs did not prevent necroptotic cell death at all in isolated macrophages but reduced inflammatory gene expression. In ApoE^−/−^ mice, this led to smaller atherosclerotic plaques and lower levels of inflammatory cytokines [8]. Together, these studies stress the complex involvement of RIPK1 either as a pro-survival scaffold or as an active kinase in atherosclerosis, which is even further complicated depending on the stage of plaque development. Karunakaran et al. hypothesized that RIPK1 is mainly involved in earlier stages of plaque development, hence their studies cover a period of only 8–10 weeks of western diet (WD), as opposed to the more advanced plaques that were obtained after 16 weeks WD in the present study.

ApoE^−/−^ RIPK1^S25D/S25D^ mice were characterized by increased levels of glycosaminoglycans both in the plaque and the tunica media, suggesting there is a general change in vascular extracellular matrix (ECM) that is not limited to plaques. Since VSMCs are the main producer of ECM components, it is tempting to propose that a change in VSMC phenotype is involved. Most likely, the RIPK1^S25D/S25D^ mutation promotes a switch in VSMCs from a contractile to synthetic phenotype so that more ECM is produced. Elevated glycosaminoglycan levels have high lipoprotein binding capacity, thereby contributing to plaque expansion. Blood vessel properties were also affected by the RIPK1^S25D/S25D^ mutation as brachiocephalic arteries of ApoE^−/−^ RIPK1^S25D/S25D^ mice were dilated. This explains why significantly larger plaques did not lead to a significant increase in stenosis in ApoE^−/−^ RIPK1^S25D/S25D^ mice. Vessel dilation combined with turbulent blood flow (due to plaques) point towards positive vascular remodeling, as first described by Glagov [37,38]. As mentioned above, RIPK1 kinase activity is linked to TLR4 signaling in response to DAMPs. TLR4 signaling is involved in atherogenesis and is known to contribute to expansive arterial remodeling [30,33,34]. These observations may link RIPK1 to the observed vessel changes, but the exact mechanism remains to be elucidated.

Clearly, transgenic and knock-out models of RIPK1 should be applied with caution, given the multitude of regulatory events that may be affected [39]. Therefore, the mono-selective, new generation RIPK1 kinase inhibitor GSK’547 was included in the present study to treat atherosclerotic ApoE^−/−^ Fbn1^C1039G+/−^ mice, which is a model of advanced, human-like atherosclerosis. By introducing the ApoE^−/−^ Fbn1^C1039G+/−^ model, we did not aim to directly compare the pharmacological approach with the genetic study in ApoE^−/−^ RIPK1^S25D/S25D^ mice. Instead, we prefer ApoE^−/−^ Fbn1^C1039G+/−^ mice for pharmacological studies because signs of necroptosis mainly occur in the more advanced stages of atherosclerosis. Indeed, the expression of phosphorylated MLKL is increased in advanced human fibroatheroma as opposed to early lesions [4]. Moreover, RIPK3 deletion in LDLr^−/−^ mice resulted in smaller plaques and less necrosis in the aortic root as compared to LDLr^−/−^ RIPK3^+/+^ controls after 16 weeks WD but not in earlier plaques after 8 weeks WD [3]. In the present study, we confirmed that expression levels of necroptosis proteins MLKL, RIPK1, and RIPK3 as well as the phosphorylation of MLKL increase during the growth of the plaque and the development of the necrotic core in ApoE^−/−^ Fbn1^C1039G+/−^ mice. Indeed, Western blot analysis showed very weak bands of P-MLKL, MLKL, and RIPK1 at baseline, and quantification of these samples should be interpreted cautiously. However, clear bands were observed after 12 and, especially, after 24 weeks WD, underlining the occurrence of necroptosis. Treatment with GSK’547 was limited to 20 weeks WD because the survival rate of ApoE^−/−^ Fbn1^C1039G+/−^ mice can drop below 50% after 20–25 weeks on WD due to myocardial infarction and stroke [28,40], and because 20 weeks WD was reported to suffice to induce an advanced plaque phenotype in ApoE^−/−^ Fbn1^C1039G+/−^ mice [27,28].

Although GSK’547 efficiently inhibited necroptosis in vitro, this effect could not be confirmed in plaques of ApoE^−/−^ Fbn1^C1039G+/−^ mice. On the contrary, plaques of GSK’547-treated mice showed higher expression levels of P-MLKL than untreated controls. Plaques contain different necroptosis ligands such as oxLDL, TNFα, and other DAMPs, and some of them are able to bypass RIPK1 kinase activity for necroptosis induction [41,42]. Changes in the plaque area and composition were not observed after GSK’547 treatment, yet GSK’547-treated ApoE^−/−^ Fbn1^C1039G+/−^ mice showed increased TUNEL and cleaved caspase 3 positivity in plaques, suggesting a switch to RIPK1 kinase-independent apoptosis as compared to untreated controls. Initially, apoptosis is preferred over necrotic cell death in atherosclerotic plaques as long as apoptotic cells are efficiently cleared, a process called efferocytosis. However, efferocytosis is impaired in advanced atherosclerosis, which results in accumulation and secondary necrosis of apoptotic bodies, contributing to plaque progression and growth of the necrotic core [7,43,44,45]. According to a recent study, GSK’547 treatment has a stage-dependent impact on atherogenesis. GSK’547 alleviates systemic inflammation in the early stages of atherosclerosis and reduces the plaque area after 2 weeks of treatment but exacerbates plaque formation after 4 weeks of treatment. Long-term GSK’547 treatment promotes macrophage accumulation and foam cell formation by upregulating the expression of several lipid metabolism-related genes. Moreover, GSK’547 inhibits ApoA1 synthesis in the liver and reduces plasma HDL levels, which contribute to plaque development [31].

In conclusion, in vitro inhibition of RIPK1 kinase activity is efficient in macrophages with a RIPK1^S25D/S25D^ mutation. However, plaque necrosis is not changed in ApoE^−/−^ RIPK1^S25D/S25D^ mice and plaque progression is even worse as compared to control mice. The increased plaque size is mainly due to increased deposition of ECM components, although a relationship between RIPK1 kinase inhibition, ECM modifications, and vascular remodeling remains to be elucidated. Pharmacological inhibition of RIPK1 kinase with GSK’547 in ApoE^−/−^ Fbn1^C1039G+/−^ mice did not alter the plaque area and composition but induced a transition to apoptosis. Therefore, without directly comparing the genetic and pharmacological studies, it can be concluded that targeting RIPK1 kinase activity is not an ideal approach to prevent plaque progression and plaque destabilization.

## Supplementary Materials

The following supporting information can be downloaded at: https://www.mdpi.com/article/10.3390/biomedicines10051016/s1, Figure S1: Leukocyte subsets in ApoE^−/−^ RIPK1^S25D/S25D^ mice; Figure S2: Leukocyte subsets in GSK’547-treated ApoE^−/−^ Fbn1^C1039G+/−^ mice.
