# Supplementary material for: The Impact of RIPK1 Kinase Inhibition on Atherogenesis: A Genetic and a Pharmacological Approach
Source: Biomedicines. 2022 Apr 28;10(5):1016. doi: 10.3390/biomedicines10051016 (PMC9138372; doi:10.3390/biomedicines10051016)

FIGURE S1: leukocyte subsets in ApoE$^{-/-}$ RIPK1$^{S25D/S25D}$ mice

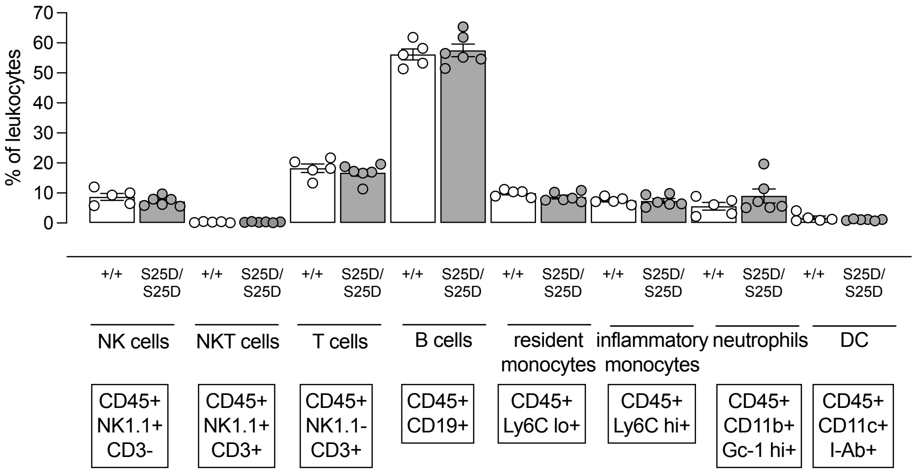

FIGURE S2: leukocyte subsets in GSK’547- treated ApoE$^{-/-}$ Fbn1$^{C1039G+/-}$ mice

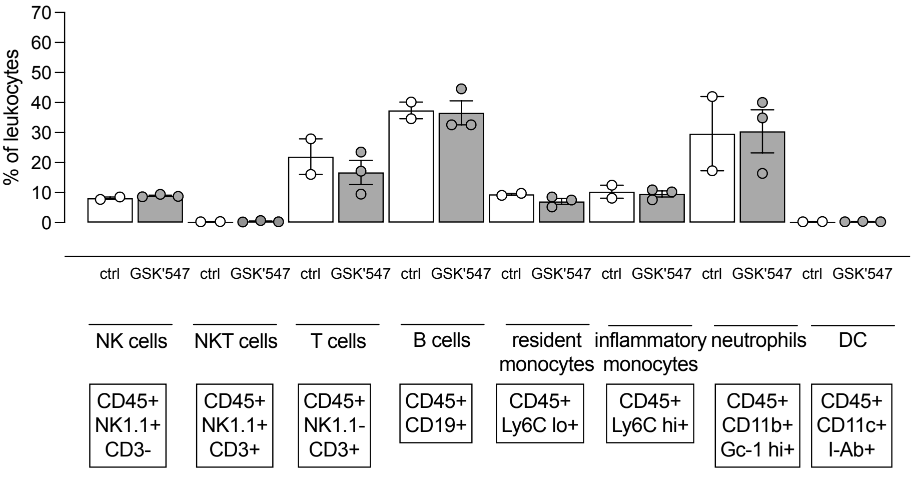

Supplement: Supplementary file 1 [file biomedicines-10-01016-s001.zip › Figures S1 and S2.pdf]
